# Genetically predicted body fat mass and distribution with diabetic kidney disease: A two-sample Mendelian randomization study

**DOI:** 10.3389/fgene.2022.872962

**Published:** 2022-09-29

**Authors:** Min Wang, Xin Li, Hang Mei, Zhao-Hui Huang, Yue Liu, Yong-Hong Zhu, Tian-Kui Ma, Qiu-Ling Fan

**Affiliations:** ^1^ Department of Nephrology, First Hospital of China Medical University, Shenyang, Liaoning, China; ^2^ Department of Nephrology, Fourth Hospital of China Medical University, Shenyang, Liaoning, China

**Keywords:** diabetic kidney disease, body mass index, body fat mass, body fat mass distribution, Mendelian randomization

## Abstract

The aim of this study is to apply a Mendelian randomization (MR) design to investigate the potential causal associations between the body mass index (BMI), body fat mass such as trunk fat mass and waist circumference (WC), and diabetic kidney disease (DKD). A two-sample MR study was conducted to obtain exposure and outcome data from previously published studies. The instrumental variables for BMI, trunk fat mass, and WC were selected from genome-wide association study datasets based on summary-level statistics. The random-effects inverse-variance weighted (IVW) method was used for the main analyses, and the weighted median and MR-Egger approaches were complementary. In total, three MR methods suggested that genetically predicted BMI, trunk fat mass, and WC were positively associated with DKD. Using IVW, we found evidence of causal relationships between BMI [odds ratio (OR) = 1.99; 95% confidence interval (CI), 1.47–2.69; *p* = 7.89 × 10^−6^], trunk fat mass (OR = 1.80; 95% CI, 1.28–2.53; *p* = 6.84 × 10^−4^), WC (OR = 2.48; 95% CI, 1.40–4.42; *p* = 1.93 × 10^−3^), and DKD. MR-Egger and weighted median regression also showed directionally similar estimates. Both funnel plots and MR-Egger intercepts showed no directional pleiotropic effects involving the aforementioned variables and DKD. Our MR analysis supported the causal effect of BMI, trunk fat mass, and WC on DKD. Individuals can substantially reduce DKD risk by reducing body fat mass and modifying their body fat distribution.

## Introduction

Approximately 40% of patients with diabetes develop diabetic kidney disease (DKD) and is the main cause of chronic kidney disease (CKD) worldwide (Alicic et al., 2017; Tuttle et al., 2021). DKD and its complications, including diabetes, heart failure, and obesity, are interrelated conditions that increase the risk of kidney failure and cardiovascular mortality and increase the cost of healthcare (Li et al., 2020). Therefore, modulating the risk factors for DKD has an important role in reducing the risk of CKD and its comorbid conditions. Obesity is associated with a high risk for both diabetes and cardiovascular complications (Melmer et al., 2018). The risk largely depends on the distribution of adipose tissue (Santilli et al., 2017). Trunk fat and waist circumference (WC) are associated with type 2 diabetes, cardiovascular disease, and multiple metabolic risk factors (Xu et al., 2015; Yang et al., 2019; Yang et al., 2021). However, these findings may have been confounded by unmeasured confounding risk factors, including objectively measured physical activity, cardiorespiratory fitness, or other underlying medical conditions that were not captured appropriately. Consequently, it is unclear whether the observed associations are causal.

Mendelian randomization (MR) is an epidemiological approach designed to evaluate causality that utilizes the principle that genotypes are free from confounding and reverse causation bias (Davey Smith and Hemani, 2014; Davies et al., 2018; Liu et al., 2021). It resembles the conditions of randomized controlled trials (RCTs), although MR may be performed retrospectively (Supplementary Figure S1). Given that all inherited genetic variants that occur prior to disease onset were determined at conception, MR has recently emerged as a tool to assess causal relationships (Emdin et al., 2017; Tan et al., 2022).

In this study, we examined whether the body mass index (BMI), trunk fat mass, and WC were causally associated with increased DKD risk using a two-sample summary MR.

## Methods

### Data sources

The summary data from published studies for this study were approved by the institutional review committee (Lawlor, 2016; Richmond et al., 2016). Therefore, further sanctions were not required. A two-sample MR was performed to evaluate the causal effect of BMI, trunk fat mass, and WC on the risk of DKD. The genome-wide association study (GWAS) summary statistics datasets used in this study were obtained from the Genetic Investigation of Anthropometric Traits (GIANT) for BMI, Neale Lab for trunk fat mass, and GIANT for WC. These single nucleotide polymorphisms (SNPs) were identified in the European population only when they reached a genome-wide significance level (*p* < 5 × 10^−8^). These variants were defined as independent based on a low correlation (*R* ^2^ < 0.001) in HapMap22 or the 1000 Genomes Project data (Genomes Project et al., 2010). Corresponding data for DKD were obtained from a global research project in Europe, which is available at Trait: Diabetic nephropathy-IEU OpenGWAS project (mrcieu.ac.uk). We obtained β-coefficients and standard errors for each allele association of each SNP and all exposures and outcomes from these data sources.

### Statistical analysis

Because individual-level GWAS data were not available, we used the recently rapidly expanding application tool of two-sample MR analyses to evaluate the causal effect of body fat mass and distribution on DKD, as previously demonstrated (Burgess et al., 2013). Horizontal pleiotropy, in which genetic variants affect outcomes through a pathway other than exposure alone, violates the assumption of MR and may lead to bias in causal estimation. To avoid this, the inverse-variance weighted (IVW), weighted median, and MR-Egger methods were applied in our study (Burgess et al., 2013; Tan et al., 2021a).

Each analytical method is based on different models of horizontal pleiotropy. Our comparison of all three results shows that the consistency of the aforementioned methods can deliver more reliable results. Moreover, two-tailed tests were used for all statistical analyses. All statistical analyses were performed using R v.4.1.23 (http://www.r-project.org) and MR software package: TwoSampleMR v.0.5.6 (Broadbent et al., 2020).

## Results

### Genetic instrumental variables

**FIGURE 1:**
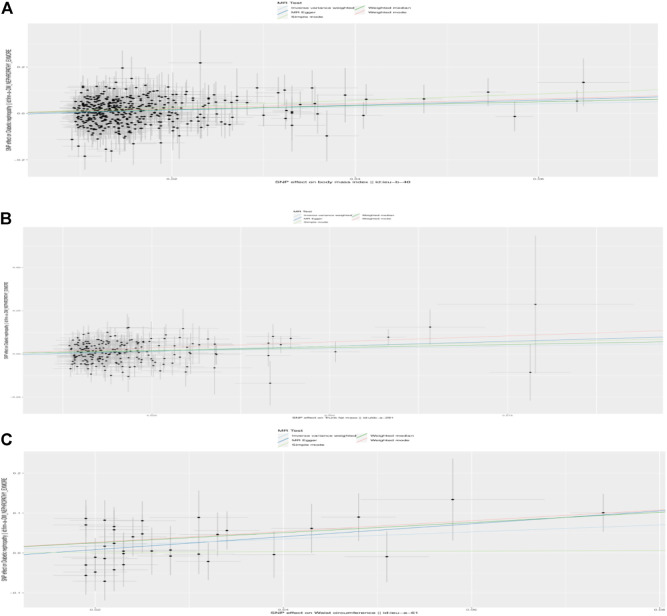
Scatterplot to visualize causal effect of the body mass index (BMI), trunk fat mass, and WC on DKD. **(A)** Causal effect of BMI on DKD. **(B)** Causal effect of trunk fat mass on DKD. **(C)** Causal effect of WC on DKD. The slope of the straight line indicates the magnitude of the causal association. IVW, inverse-variance weighted; MR, Mendelian randomization.

**FIGURE 2:**
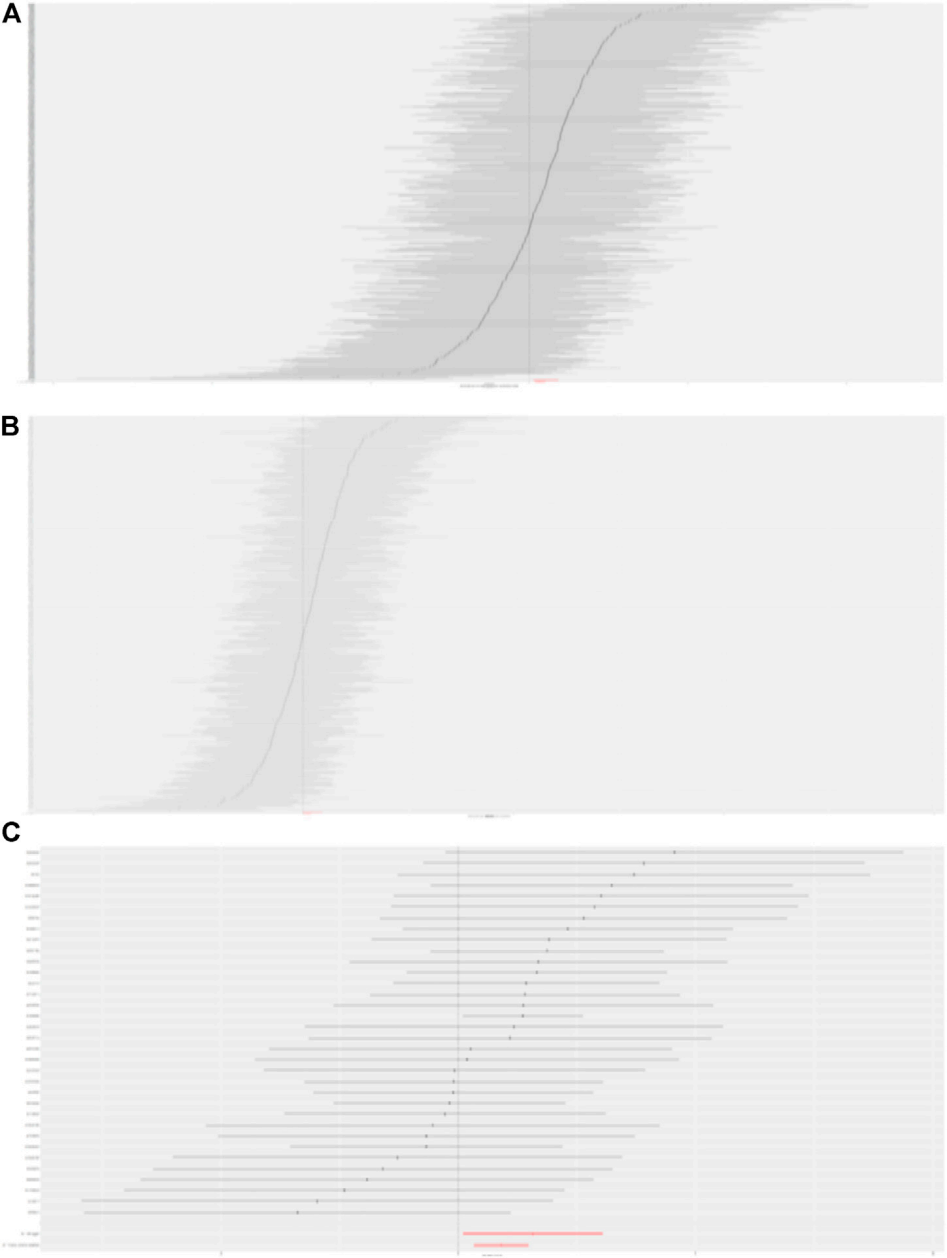
Forest plot to visualize causal effect of each SNP on DKD. **(A)** Causal effect of BMI on DKD. **(B)** Causal effect of trunk fat mass on DKD. **(C)** Causal effect of WC on DKD.

In total, 441, 214, and 34 instrumental variables (IVs) were chosen for BMI, trunk fat mass, and WC on DKD, respectively. Detailed information concerning the IVs used in our study is presented in Supplementary Tables S1–S3. The causal effects of each genetic variant on DKD are shown in Figures 1, 2.

### Mendelian randomization analysis for body mass index, trunk fat mass, and waist circumference

**FIGURE 3:**
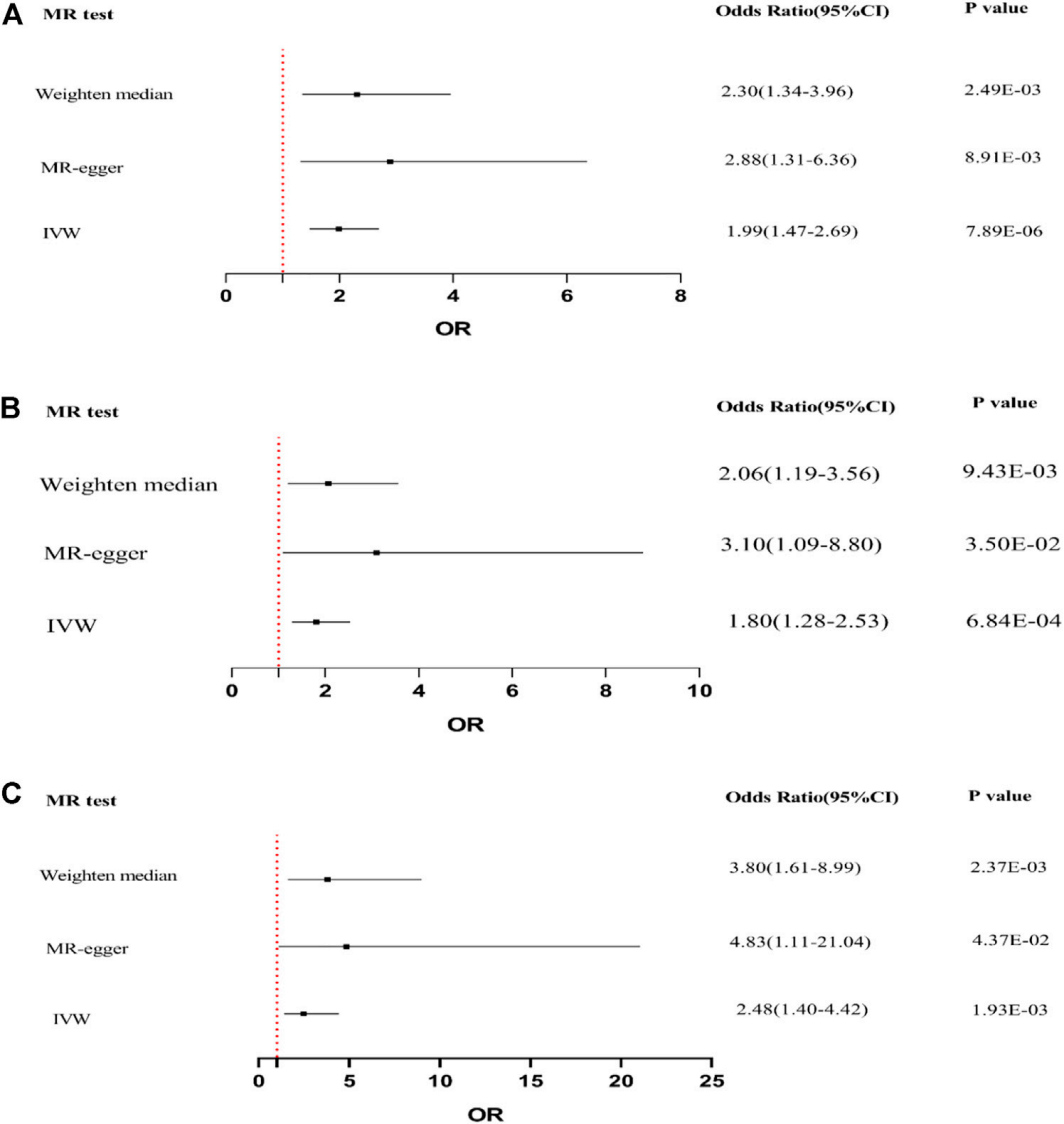
Forest plot to visualize causal effect of BMI, trunk fat mass, and WC on the risk of DKD by three methods. **(A)** Causal effect of BMI on DKD. **(B)** Causal effect of trunk fat mass on DKD. **(C)** Causal effect of WC on DKD.

IVW, MR-Egger, and weighted median regression were used to evaluate causal associations between genetically predicted BMI, trunk fat mass, WC, and DKD (Figure 3). Furthermore, three MR methods consistently supported the causal effect of higher BMI, trunk fat mass, and WC on an elevated DKD risk. BMI was significantly positively associated with DKD [IVW odds ratio (OR) per SD increase in BMI = 1.99 (95% CI, 1.47–2.69), *p* = 7.89 × 10^−6^; trunk fat mass = 1.80 (95% CI, 1.28–2.53), *p* = 6.84 × 10^−4^; and WC = 2.48 (95% CI, 1.40–4.42), *p* = 1.93 × 10^−3^]. MR-Egger and weighted median regression also showed directionally similar estimates [MR-Egger OR per SD increase in BMI, 2.88 (95% CI, 1.31–6.36), *p* = 8.91 × 10^−3^; trunk fat mass, 3.10 (95% CI, 1.09–8.80), *p* = 3.50 × 10^−2^; WC, 4.83 (95% CI, 1.11–21.04), *p* = 3.50 × 10^−2^; weighted median OR per SD increase in BMI, 2.30 (95% CI, 1.34–3.96), *p* = 2.49 × 10^−3^; and trunk fat mass, 2.06 (95% CI, 1.19–3.56), *p* = 9.43 × 10^−3^].

### Analysis of horizontal pleiotropy

**FIGURE 4:**
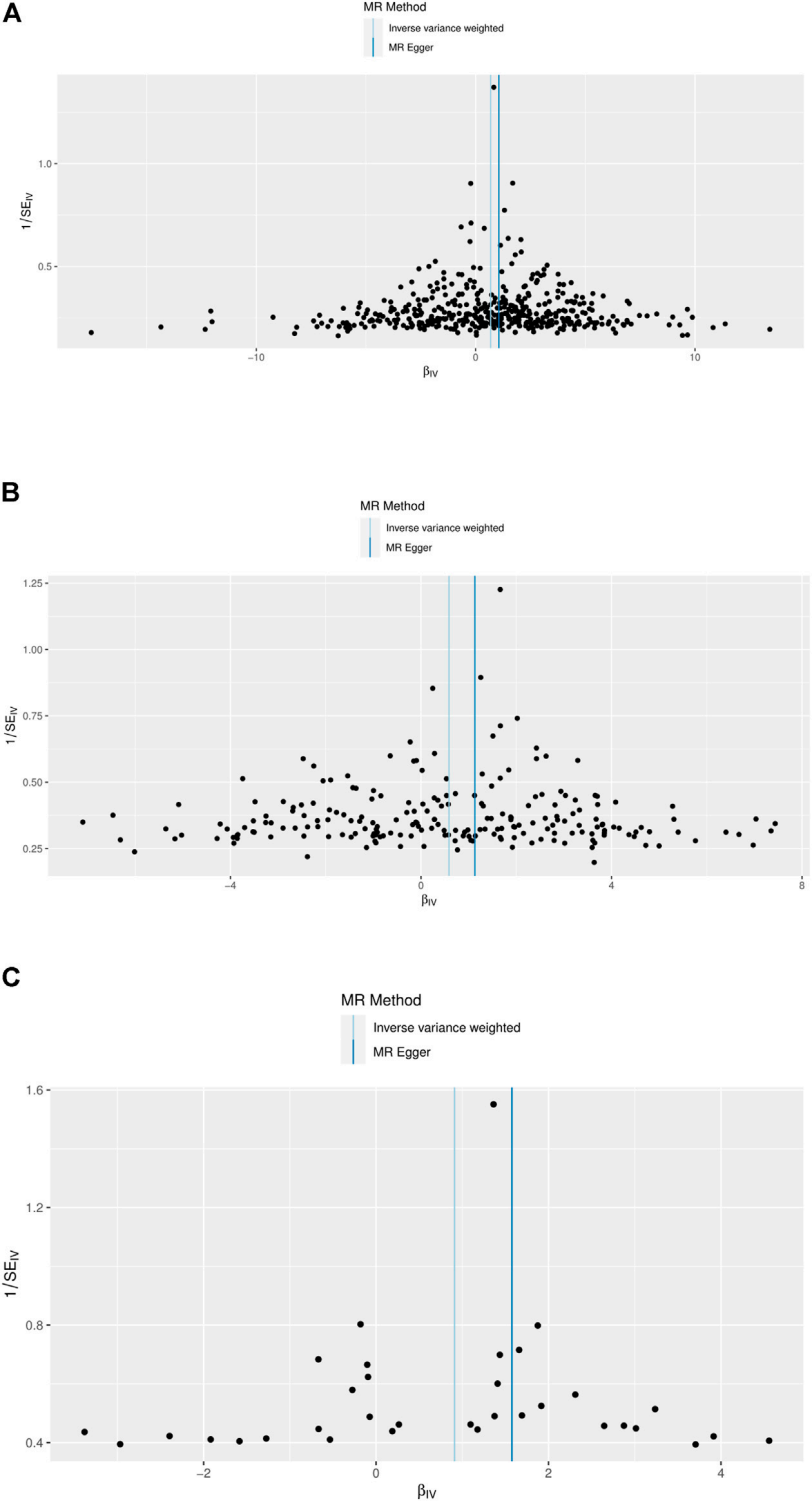
Funnel plots to visualize overall heterogeneity of MR estimates for the effect of BMI, trunk fat mass, and WC on DKD. **(A)** Causal effect of BMI on DKD. **(B)** Causal effect of trunk fat mass on DKD. **(C)** Causal effect of WC on DKD. IVW, inverse-variance weighted; MR, Mendelian randomization.

Funnel plots indicated the existence of directional horizontal pleiotropy for each SNP. The causal effect of our funnel plots was roughly symmetrical (Figure 4). MR-Egger intercepts were also conducted, which revealed no evidence of statistically significant differences in directional pleiotropy for DKD in our study (BMI, *p* = 0.319; trunk fat mass, *p* = 0.320; WC, *p* = 0.293). These results suggest that no directional pleiotropic effects are present in our study.

## Discussion

In this population-based cohort study, MR was conducted to investigate the causal impact of BMI, trunk fat mass, and WC on DKD. In agreement with previous observational studies, our findings suggested that genetically mediated BMI, trunk fat mass, and WC are causally associated with an increased risk of DKD, where a 1 SD increase in the above-mentioned genetic IVs conferred a 99%, 80%, or 148% increased risk of DKD in the European population, respectively.

Each SD with higher genetic BMI (1.99; 1.47–2.69), trunk fat mass (1.80; 1.28–2.53), and WC (2.48; 1.40–4.42) was related to increased DKD risk. Human epidemiological studies suggest that obesity is a major cause of DKD, and the growing incidence of obesity contributes to diabetes associated with CKD prevalence (Winocour, 2018; Yang et al., 2020). An MR analysis in individuals of European ancestry indicated that each SD in higher BMI conferred an increased risk of DKD (1.33; 1.17–1.51). Our results are very similar to those of previous observational studies and MR analyses (Todd et al., 2015; Wan et al., 2020; Polemiti et al., 2021). However, certain research do not support this conclusion (Keane et al., 2003; Huang et al., 2014). A study showed that a high BMI (≥25 kg/m^2^) was a protective factor against worsening renal function in patients with type 2 diabetes mellitus complicated by CKD at stages 3 or 4 (Huang et al., 2014). Inconsistent results in observational studies can be explained by reverse causal bias or may be confounded by unmeasured confounding risk factors. MR can compensate for the limitations of conventional epidemiological studies and can be used to assess causal relationships. However, only one method was utilized in the previous MR (Todd et al., 2015). The consistency of the three methods we used to render our study more reliable.

Accumulation of trunk fat mass is associated with higher glucose levels, post-load glucose levels, and diabetes risk in observational epidemiological studies (Snijder et al., 2004; Tatsukawa et al., 2018). In addition, it is correlated with both visceral and subcutaneous fat accumulation as evaluated by computed tomography and is considered an inflammatory marker in non-dialyzed CKD patients (Axelsson et al., 2004; Sanches et al., 2008). Choi et al. (2017) confirmed that an increase of 1 kg in trunk fat mass is associated with a 15% increase in men with DM and a 19% increase in women after adjustment for several confounding factors. Using the MR method in current European populations, our results are in accordance with those of the above study (1.80; 1.28–2.53). WC has mostly been employed as a surrogate marker of abdominal adiposity. A number of studies involving CKD and non-CKD subjects have confirmed a strong correlation between WC and trunk fat, as evaluated using dual-energy X-ray absorptiometry (Orsatti et al., 2010; Bazanelli et al., 2012). Xu et al. (2015) suggested that a higher WC is associated with greater odds of developing CKD in type 2 diabetes mellitus (1.019 and 1.002–1.006). Our study provided by MR arrives at a similar conclusion: each SD with higher genetic WC (2.48; 1.40–4.42) was found to have a positive causal effect on DKD. These results revealed that higher overall and body fat distributions are causal risk factors for DKD in European populations. Moreover, our MR studies suggested that the causal effect of WC on DKD risk was greater than that of overall obesity. Obesity can stimulate the formation of lipid metabolites, cytokines, and hormones, which involves changes in the insulin signaling pathway and accelerates progression of insulin resistance.

Obesity is a condition in which the number and size of adipocytes increase, which further increases the total fat mass (Balsan et al., 2015). Adiponectin, which is mainly secreted by adipocytes, is considered to have anti-inflammatory, anti-atherosclerotic, and insulin-sensitizing properties. It regulates energy homeostasis and glucose and lipid mechanisms *via* the activation of adenosine monophosphate-activated protein kinases (Yamauchi et al., 2002). Snijder et al. (2009) revealed that trunk fat is negatively correlated with adiponectin levels. This may explain the adverse effect of trunk fat mass on DKD. Moreover, the causal effects of overall obesity, trunk fat mass, WC on DKD, and WC were slightly greater than those of generalized obesity. Thus, we emphasize that both the mass and distribution of body fat play a causal role in DKD.

Observational studies are more susceptible to reverse causality or confounding than the MR analysis, which can provide the best evidence for assessing causal relationships involving BMI, trunk fat mass, WC, and DKD etiology. Statistical differences were evaluated using the two-sample MR approach. To minimize potential pleiotropy, three different methods were performed to evaluate directional pleiotropy. A strength of this study was that the causal effects of BMI, trunk fat mass, and WC on DKD risk are robust and unbiased, owing to the consistency of the three methods. Another strength is that we simultaneously assessed the relationship between different risk factors and DKD, the most common micro-vascular complication of diabetes. Therefore, we were able to investigate the differences across disease risk factors.

One potential limitation of our study is that only European descent were recruited. However, regarding disease heterogeneity, studies have demonstrated different genetic profiles in different cohorts of patients, which are attributed to ethnic diversity, different risk factors, and different epigenetic profiles. Therefore, the reliability of the causal associations should be verified in other non-European populations (Tan et al., 2021b).

In short, overall and body fat distributions have a causal effect on DKD risk, and WC may exert greater effects. These results reveal that individuals can substantially reduce DKD risk for diabetes by reducing body fat mass and modifying body fat distribution.

## Data Availability

The original contributions presented in the study are included in the article/Supplementary Material; further inquiries can be directed to the corresponding author.

## Supplementary material

SUPPLEMENTARY FIGURE S1 Schematic representation of an MR analysis. We selected the genetic variants associated with risk factors that can be used to evaluate the causal effect of risk factors on DKD obtained from a large cohort of European population.
