# Supplementary figures and images for: Genetically predicted body fat mass and distribution with diabetic kidney disease: A two-sample Mendelian randomization study
Source: Front Genet. 2022 Sep 29;13:872962. doi: 10.3389/fgene.2022.872962 (PMC9557077; doi:10.3389/fgene.2022.872962)

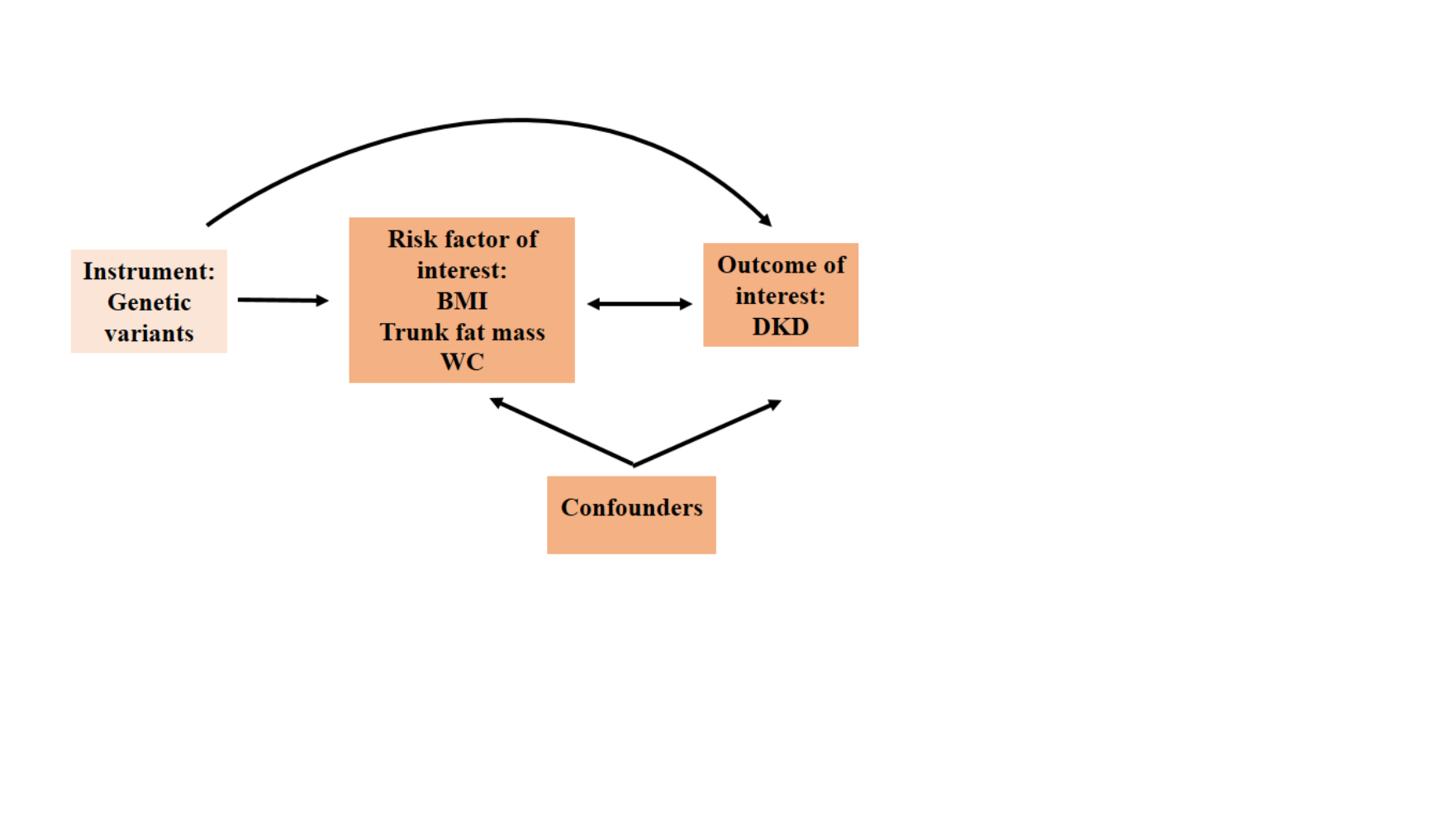

Supplement: Supplementary file 2 [file Image1.TIF]
