# Supplementary material for: Genetically predicted body fat mass and distribution with diabetic kidney disease: A two-sample Mendelian randomization study
Source: Front Genet. 2022 Sep 29;13:872962. doi: 10.3389/fgene.2022.872962 (PMC9557077; doi:10.3389/fgene.2022.872962)
Supplement: Supplementary file 3 [file DataSheet1.PDF]

Supplementary Table S1. List of genetic instruments for BMI by each instrumental SNPs.

No.	SNP	chr	EA	OA	EAF	**β**	SE
1	rs6595205	5	C	G	0.5403	0.0022	0.0469
2	rs6772756	3	A	G	0.641	0.0207	0.0491
3	rs2051559	4	C	T	0.1074	0.0324	0.0765
4	rs1656377	3	C	T	0.5586	0.033	0.0469
5	rs2643452	4	A	T	0.4055	0.0603	0.0476
6	rs4148155	4	A	G	0.92561	-0.1266	0.0905
7	rs12416812	11	A	G	0.5646	0.0716	0.0472
8	rs2875762	6	G	C	0.8892	0.0811	0.075
9	rs38314	7	A	G	0.5312	-0.0389	0.0469
10	rs16849710	1	G	A	0.527	-0.0121	0.0467
11	rs4653017	1	C	T	0.3304	0.0165	0.0496
12	rs13021737	2	G	A	0.838	-0.0129	0.0635
13	rs2861683	2	A	C	0.5844	0.0332	0.0475
14	rs1445652	2	G	A	0.8629	-0.0222	0.0686
15	rs4414033	1	G	A	0.4298	-0.0183	0.0474
16	rs1296328	4	A	C	0.5192	0.0246	0.0469
17	rs3806572	2	A	G	0.2429	0.0038	0.0544
18	rs17424296	5	G	A	0.674	-0.0062	0.05
19	rs9294260	6	A	G	0.5062	-0.0061	0.0468
20	rs2033529	6	G	A	0.3277	0.0822	0.0498
21	rs3749897	6	T	C	0.4498	0.0074	0.0469
22	rs486359	6	G	C	0.5008	-0.0028	0.0467
23	rs7138803	12	A	G	0.3807	-0.0077	0.0483
24	rs2608703	12	C	A	0.5636	0.0453	0.0471
25	rs1477199	16	G	A	0.1137	-0.0163	0.0739
26	rs208015	17	T	C	0.1074	0.0425	0.075
27	rs1365466	18	C	T	0.1958	0.0551	0.059
28	rs17710386	18	T	C	0.6039	-0.0941	0.0482
29	rs3935648	17	G	C	0.1768	0.074	0.0614
30	rs7998796	13	G	A	0.3711	0.0365	0.0484
31	rs1112613	13	A	G	0.1969	0.0341	0.0584
32	rs13132853	4	G	A	0.378	-0.0034	0.0484
33	rs538579	3	C	G	0.2623	-0.0579	0.0534
34	rs6785245	3	C	T	0.3498	0.0326	0.0489
35	rs946824	1	C	T	0.8527	0.0627	0.0656
36	rs4639527	2	G	A	0.3745	0.0789	0.0485
37	rs1522569	4	T	G	0.9152	0.0739	0.0848
38	rs7683836	4	A	G	0.4765	0.0345	0.0466

39	rs930295	2	A	C	0.1284	0.0453	0.0692
40	rs9845966	3	G	T	0.489	-0.0052	0.0468
41	rs2246012	6	C	T	0.2286	0.0645	0.0559
42	rs7819514	8	G	A	0.6163	0.0647	0.0486
43	rs1948080	9	G	T	0.3029	-0.0468	0.0511
44	rs3800229	6	G	T	0.3638	-0.0425	0.0488
45	rs200810	6	C	T	0.3911	-0.0013	0.0483
46	rs156201	6	C	G	0.6241	0.0068	0.0485
47	rs998732	19	A	G	0.8999	0.1212	0.0778
48	rs10858334	9	C	G	0.924	0.0896	0.0879
49	rs3977755	10	C	T	0.7923	0.0453	0.0578
50	rs774246	7	G	A	0.119	0.0429	0.0726
51	rs8071182	17	A	G	0.1521	-0.0415	0.066
52	rs889398	16	T	C	0.426	0.0081	0.0476
53	rs1836303	15	A	G	0.6784	-0.0347	0.0502
54	rs217671	14	A	G	0.7897	-0.0442	0.0573
55	rs10492229	12	C	T	0.7276	0.0764	0.0525
56	rs9926784	16	C	T	0.1735	0.0454	0.0615
57	rs852056	20	C	T	0.7671	0.1538	0.0554
58	rs11150911	18	A	C	0.3081	0.0136	0.0505
59	rs895330	19	C	G	0.8346	0.0276	0.0629
60	rs10953740	7	G	A	0.5189	0.0592	0.0468
61	rs3800637	7	C	T	0.3385	0.07	0.0497
62	rs8181823	13	A	C	0.2715	-0.0089	0.0524
63	rs339991	15	G	A	0.6159	-0.0002	0.0478
64	rs1535660	9	C	T	0.8894	0.0167	0.0748
65	rs1048932	11	C	A	0.5678	0.0014	0.0473
66	rs11105839	12	A	T	0.3621	0.0765	0.0487
67	rs4842491	12	T	C	0.6941	-0.0229	0.0509
68	rs6591407	11	A	C	0.1479	-0.0753	0.067
69	rs2284746	1	C	G	0.5096	-0.0407	0.0465
70	rs12041258	1	T	C	0.7626	0.0175	0.0546
71	rs17001561	4	G	A	0.8853	0.044	0.0733
72	rs10811871	9	G	A	0.3786	0.0318	0.0481
73	rs10742752	11	T	C	0.4282	0.033	0.0475
74	rs4937870	11	G	A	0.4164	-0.0024	0.0477
75	rs10938397	4	A	G	0.5255	0.0214	0.0468
76	rs2744974	6	C	T	0.6664	0.0024	0.0503
77	rs3844598	5	A	G	0.4373	-0.0015	0.0471
78	rs2479958	13	G	A	0.5133	0.0163	0.0468
79	rs9571687	13	A	C	0.2267	-0.0607	0.0555
80	rs11173522	12	C	A	0.8084	0.0277	0.0593
81	rs10132280	14	C	A	0.6489	0.0663	0.049

82	rs7037266	9	A	C	0.4053	0.0214	0.0476
83	rs754635	3	G	C	0.8998	-0.0087	0.078
84	rs7551507	1	C	T	0.509	-0.0073	0.0465
85	rs8090983	18	G	A	0.3434	-0.0475	0.0492
86	rs4813619	20	G	T	0.4405	0.0748	0.0471
87	rs4012234	20	G	T	0.6489	-0.0265	0.0492
88	rs17724992	19	A	G	0.7773	0.0155	0.0561
89	rs769449	19	G	A	0.8353	-0.0363	0.0637
90	rs3736485	15	G	A	0.5877	0.1618	0.0474
91	rs12888545	14	A	G	0.765	-0.0569	0.0549
92	rs1285997	14	G	C	0.7362	-0.0649	0.0527
93	rs4783830	16	G	A	0.678	0.0041	0.0501
94	rs9304665	19	A	T	0.7191	-0.0332	0.0521
95	rs4906908	15	G	T	0.5097	-0.0536	0.0465
96	rs9478671	6	G	A	0.1746	0.0753	0.0613
97	rs3754963	2	A	T	0.6943	-0.0582	0.0511
98	rs17499593	2	C	G	0.8726	-0.0004	0.0695
99	rs10269783	7	A	G	0.4602	0.0324	0.047
100	rs10247983	7	G	A	0.0857	0.0744	0.0842
101	rs645040	3	G	T	0.1478	0.0466	0.0657
102	rs10920678	1	G	A	0.4752	-0.0676	0.0469
103	rs11118308	1	G	A	0.454	0.026	0.0471
104	rs10915840	1	G	A	0.7701	0.0124	0.0556
105	rs902695	2	A	G	0.4142	-0.0112	0.0472
106	rs13069244	3	A	G	0.04569	0.0007	0.113
107	rs10832778	11	G	C	0.5622	-0.0314	0.0472
108	rs11611246	12	G	T	0.786	-0.0665	0.0569
109	rs1268065	6	G	A	0.5202	0.0076	0.0466
110	rs2907948	7	G	A	0.8308	0.0277	0.0624
111	rs1064213	2	G	A	0.603	-0.0689	0.048
112	rs1993709	1	G	A	0.8603	0.0069	0.0674
113	rs10747488	1	A	C	0.6848	0.0199	0.0501
114	rs13147390	4	C	T	0.306	0.1115	0.0508
115	rs4952843	2	G	A	0.3351	-0.0289	0.0499
116	rs4954638	2	C	A	0.347	0.0071	0.0493
117	rs1528435	2	C	T	0.3606	0.0385	0.0487
118	rs7599312	2	A	G	0.3018	0.0114	0.0509
119	rs11889536	2	A	G	0.94842	0.0704	0.107
120	rs4858193	3	C	T	0.2614	-0.009	0.0531
121	rs2931434	5	C	T	0.7021	0.0312	0.0508
122	rs13184896	5	T	G	0.4392	0.0416	0.047
123	rs2423668	20	C	T	0.5649	-0.0072	0.0471
124	rs13110266	4	G	A	0.5843	0.0496	0.0474

125	rs685870	11	T	C	0.308	0.0536	0.0506
126	rs2832283	21	G	A	0.7404	0.0664	0.0537
127	rs825688	16	T	C	0.4328	-0.0515	0.0474
128	rs12936083	17	G	A	0.4138	0.021	0.0477
129	rs4986044	17	T	C	0.4961	-0.0204	0.0468
130	rs273504	19	G	A	0.4899	0.0186	0.0466
131	rs12150665	17	T	C	0.629	0.022	0.0483
132	rs872281	14	T	C	0.242	0.0215	0.0546
133	rs10962550	9	C	G	0.1722	0.04	0.0619
134	rs1260326	2	T	C	0.3524	-0.071	0.0491
135	rs17399237	2	C	T	0.616	0.046	0.0482
136	rs543874	1	G	A	0.1763	0.0625	0.0614
137	rs2943465	12	T	C	0.0772	-0.049	0.0889
138	rs4880341	10	C	T	0.4876	0.1166	0.0467
139	rs380857	9	C	A	0.1814	-0.0261	0.0609
140	rs7761673	6	T	A	0.8466	-0.0128	0.0647
141	rs2174307	9	C	G	0.4926	0.0286	0.0468
142	rs3806114	6	A	G	0.7544	-0.0369	0.0543
143	rs4757144	11	A	G	0.506	-0.0233	0.0467
144	rs7780752	7	C	T	0.2654	0.0291	0.0529
145	rs11496125	7	C	T	0.5377	-0.0123	0.0467
146	rs987237	6	A	G	0.791	0.0086	0.0575
147	rs9688431	6	T	C	0.97061	0.2178	0.1403
148	rs6461115	7	G	A	0.3068	-0.0547	0.0508
149	rs1830074	7	C	T	0.3195	0.0312	0.0502
150	rs768840	14	G	A	0.6317	0.0842	0.0485
151	rs7181498	15	C	T	0.5526	0.0323	0.0469
152	rs2367112	5	T	G	0.4715	-0.1099	0.047
153	rs11951673	5	C	T	0.6976	0.0349	0.0507
154	rs806600	5	A	G	0.5258	-0.0349	0.0468
155	rs1476322	3	G	A	0.5035	-0.0516	0.0468
156	rs6443750	3	T	C	0.2116	-0.0034	0.0584
157	rs7811342	7	C	T	0.1722	0.0247	0.0619
158	rs6448587	4	A	C	0.7734	-0.064	0.0558
159	rs13287131	9	C	T	0.2791	0.0849	0.0518
160	rs3904244	10	T	A	0.7662	-0.0514	0.0552
161	rs3764835	2	G	A	0.8143	-0.0054	0.0602
162	rs2693826	2	A	G	0.5121	-0.0247	0.0466
163	rs1472169	9	C	T	0.6922	-0.0343	0.0507
164	rs349088	11	C	A	0.3848	-0.0153	0.0486
165	rs4936175	11	C	T	0.358	-0.0055	0.0488
166	rs12364470	11	G	T	0.2251	0.0563	0.0562
167	rs1538247	6	C	T	0.3203	0.1005	0.0501

168	rs947612	6	G	A	0.2743	0.0112	0.052
169	rs262130	6	C	T	0.7957	0.0251	0.0581
170	rs9362662	6	G	A	0.5777	-0.0389	0.0472
171	rs11713193	3	A	G	0.3973	-0.0509	0.0478
172	rs10197031	2	C	T	0.3442	-0.0372	0.0493
173	rs11738695	5	C	A	0.4226	0.0489	0.0474
174	rs2600226	3	C	T	0.3642	0.0017	0.0487
175	rs33500	3	C	T	0.1862	0.0121	0.0604
176	rs7626079	3	T	C	0.3063	0.009	0.0509
177	rs657452	1	A	G	0.4272	-0.0017	0.0471
178	rs6593688	1	A	G	0.6174	-0.0294	0.048
179	rs12564992	1	A	G	0.91863	-0.0461	0.0855
180	rs11609659	12	T	C	0.7449	-0.0759	0.0534
181	rs7983065	13	T	C	0.4503	-0.0101	0.0472
182	rs9783858	18	C	T	0.475	0.1122	0.0469
183	rs1884389	20	T	C	0.4311	-0.0442	0.0471
184	rs1681740	10	A	C	0.6344	-0.0085	0.0487
185	rs6265	11	C	T	0.8448	0.0611	0.0647
186	rs9522285	13	A	G	0.4765	-0.0633	0.0468
187	rs12369179	12	T	C	0.08322	0.0021	0.0845
188	rs10518694	15	C	A	0.8929	-0.1958	0.0752
189	rs17238110	15	A	G	0.895	0.1098	0.0767
190	rs8097783	18	G	A	0.95807	0.079	0.1175
191	rs17513613	19	T	C	0.6438	0.0362	0.0492
192	rs9547153	13	A	G	0.6007	0.1406	0.0477
193	rs8047395	16	G	A	0.498	-0.0531	0.0468
194	rs7144011	14	G	T	0.763	-0.0465	0.0549
195	rs709400	14	G	A	0.3086	-0.0169	0.0504
196	rs9927848	16	C	A	0.1976	-0.0882	0.0589
197	rs2285178	22	T	C	0.6775	0.0274	0.0499
198	rs6712	22	C	G	0.133	0.0116	0.0688
199	rs138289	22	T	A	0.3716	0.0339	0.0484
200	rs12044597	1	G	A	0.5145	0.0248	0.0469
201	rs6692586	1	G	A	0.8852	-0.0375	0.0734
202	rs705217	1	G	T	0.3801	-0.0116	0.0484
203	rs4660443	1	C	T	0.7954	-0.0298	0.0581
204	rs17014375	1	G	T	0.1302	0.0307	0.0691
205	rs2235564	1	T	C	0.3641	0.0363	0.0486
206	rs10914462	1	G	A	0.3991	0.054	0.0479
207	rs6587552	1	G	A	0.7506	-0.0776	0.0536
208	rs17425707	1	T	C	0.93338	0.0136	0.094
209	rs1891216	1	G	T	0.4294	0.0323	0.0473
210	rs2791653	1	G	A	0.7155	-0.0513	0.0521

211	rs2481665	1	T	C	0.6351	0.1113	0.0486
212	rs7519259	1	A	G	0.5658	-0.0126	0.0475
213	rs12049202	1	C	T	0.7191	-0.0626	0.0521
214	rs2007231	1	T	C	0.6912	0.0615	0.0509
215	rs10733051	1	G	A	0.5719	-0.0147	0.0471
216	rs977747	1	T	G	0.3644	0.0823	0.0484
217	rs11165643	1	T	C	0.596	-0.0052	0.0476
218	rs11185111	1	G	A	0.5657	-0.0155	0.0473
219	rs7550711	1	C	T	0.94672	-0.134	0.1029
220	rs2820311	1	G	A	0.2967	-0.0182	0.0509
221	rs4358081	2	A	C	0.553	0.0173	0.047
222	rs6545714	2	A	G	0.6072	-0.0241	0.0476
223	rs1371108	2	A	C	0.2978	0.0104	0.0512
224	rs4851029	2	G	T	0.5145	0.018	0.0468
225	rs10192119	2	G	T	0.1981	-0.0642	0.059
226	rs1521527	2	C	G	0.6139	-0.0002	0.0486
227	rs10169594	2	T	C	0.6128	0.0617	0.0481
228	rs4556997	2	C	A	0.853	-0.0062	0.066
229	rs429343	2	A	G	0.467	0.1051	0.047
230	rs12328930	2	T	C	0.6084	0.0132	0.0482
231	rs3731695	2	C	T	0.4506	-0.0235	0.0468
232	rs7557796	2	T	C	0.3957	-0.0667	0.0478
233	rs4482463	2	A	C	0.9586	-0.0019	0.1178
234	rs3732084	2	T	C	0.4889	-0.0181	0.0468
235	rs2162524	2	T	C	0.7108	0.0825	0.0515
236	rs10182181	2	A	G	0.58	-0.0128	0.0474
237	rs934224	2	C	T	0.3156	0.0308	0.0501
238	rs17551974	2	C	A	0.7878	0.0558	0.057
239	rs17203016	2	G	A	0.2112	-0.0011	0.0572
240	rs2317299	2	C	T	0.5362	0.0383	0.0473
241	rs17033117	3	T	C	0.1674	0.0355	0.0621
242	rs1452075	3	C	T	0.2945	-0.0084	0.0515
243	rs1006896	3	C	A	0.8218	-0.0411	0.0612
244	rs9816226	3	T	A	0.8522	0.1007	0.0665
245	rs1552893	3	G	A	0.2285	-0.0391	0.0555
246	rs6764533	3	A	G	0.3037	0.0583	0.0508
247	rs17535749	3	G	A	0.915	-0.0271	0.0833
248	rs3772882	3	A	C	0.4431	0.0113	0.0469
249	rs7640424	3	T	C	0.4097	-0.0545	0.0474
250	rs2868975	3	A	G	0.7687	0.0287	0.0556
251	rs8192675	3	C	T	0.2679	-0.0583	0.0527
252	rs10510419	3	T	G	0.1445	0.0491	0.0663
253	rs2365389	3	C	T	0.5616	0.0692	0.0471

254	rs12629015	3	A	G	0.7417	0.0259	0.0533
255	rs1320903	3	A	G	0.2941	0.0951	0.0513
256	rs16851483	3	T	G	0.04828	-0.0966	0.1093
257	rs7615297	3	C	G	0.8372	-0.0981	0.0632
258	rs6804842	3	G	A	0.5792	0.0327	0.0474
259	rs7637852	3	G	A	0.5859	-0.1342	0.0477
260	rs2325036	3	A	C	0.5384	0.0316	0.0469
261	rs1454687	3	G	C	0.5909	0.0588	0.0476
262	rs1436344	3	G	C	0.3669	0.0446	0.0485
263	rs2124499	3	C	G	0.3408	-0.0232	0.0496
264	rs355777	3	C	G	0.4305	-0.0805	0.0474
265	rs326896	4	T	C	0.3162	-0.115	0.0504
266	rs331966	4	A	C	0.7138	-0.067	0.052
267	rs11736228	4	A	T	0.7683	0.0158	0.0554
268	rs1492767	4	T	C	0.4189	0.0234	0.0474
269	rs7685048	4	C	T	0.6119	0.0048	0.0479
270	rs10009336	4	T	C	0.1119	-0.0171	0.0737
271	rs6815910	4	T	A	0.424	-0.0477	0.0477
272	rs7694732	4	A	G	0.5426	0.0226	0.047
273	rs11945861	4	A	G	0.2251	0.1053	0.0563
274	rs1804528	4	A	G	0.4004	0.0302	0.0476
275	rs6841761	4	G	T	0.3874	0.0423	0.0481
276	rs1863652	4	A	G	0.2942	0.0721	0.0511
277	rs7730004	5	C	T	0.4293	0.014	0.0472
278	rs7715256	5	T	G	0.5907	-0.0622	0.0475
279	rs2307111	5	T	C	0.5834	0.0479	0.0476
280	rs6235	5	G	C	0.7174	0.0339	0.0518
281	rs10478110	5	C	A	0.5245	-0.0406	0.0468
282	rs7724675	5	A	G	0.2647	0.001	0.0534
283	rs7703576	5	C	T	0.3345	-0.0092	0.0495
284	rs16871902	5	G	A	0.4918	-0.0537	0.0468
285	rs7704281	5	A	G	0.95966	0.0734	0.12
286	rs1503526	5	C	T	0.5751	0.0725	0.0476
287	rs2009416	5	T	C	0.4334	-0.0758	0.047
288	rs40067	5	G	A	0.7893	-0.0167	0.0578
289	rs294704	5	T	G	0.7663	0.0922	0.0553
290	rs4518345	5	A	G	0.7585	-0.0678	0.0548
291	rs10942267	5	G	A	0.2444	0.0459	0.0539
292	rs17056301	5	C	T	0.2206	0.0237	0.0562
293	rs189843	5	G	C	0.3884	0.0241	0.0481
294	rs17663412	5	C	A	0.92007	-0.0415	0.0866
295	rs7730898	5	A	G	0.6428	0.0378	0.0488
296	rs6556301	5	T	G	0.3817	-0.0275	0.0487

297	rs2228213	6	G	A	0.6551	0.0608	0.049
298	rs9367368	6	T	C	0.6112	0.0238	0.0482
299	rs9379827	6	A	C	0.2367	-0.0143	0.0548
300	rs9370261	6	T	C	0.05142	-0.0035	0.1056
301	rs1885728	6	A	G	0.6786	-0.0179	0.05
302	rs1327259	6	G	A	0.3835	-0.0324	0.0481
303	rs2357760	6	A	G	0.6725	-0.0404	0.0497
304	rs1266874	6	A	G	0.5652	-0.0162	0.0469
305	rs901630	6	C	T	0.6866	-0.0542	0.0502
306	rs17789218	6	C	T	0.2091	0.0705	0.0576
307	rs13191362	6	A	G	0.93872	0.0358	0.0966
308	rs17207196	7	C	T	0.4842	-0.0331	0.0468
309	rs13240600	7	A	G	0.7737	0.0096	0.0559
310	rs7788008	7	G	A	0.6005	0.0645	0.0478
311	rs4722398	7	T	C	0.93038	0.1195	0.0911
312	rs10243319	7	T	C	0.5598	0.0394	0.0471
313	rs215634	7	G	A	0.7356	0.0475	0.053
314	rs2283093	7	T	C	0.8154	0.0259	0.0602
315	rs2543132	8	C	G	0.856	0.069	0.0668
316	rs12546578	8	T	A	0.2408	-0.0125	0.0554
317	rs12680842	8	G	A	0.4152	0.0128	0.0477
318	rs12675063	8	T	A	0.9345	-0.1507	0.0936
319	rs4072917	8	G	A	0.5052	0.0225	0.0467
320	rs7844647	8	T	C	0.7576	0.0416	0.0547
321	rs13250058	8	G	T	0.3285	0.0713	0.0498
322	rs6985109	8	G	A	0.633	-0.0135	0.0484
323	rs6471941	8	A	G	0.3238	-0.0399	0.05
324	rs1431659	8	A	G	0.261	0.0637	0.0532
325	rs2694047	8	G	A	0.7068	-0.0538	0.0514
326	rs13263601	8	C	A	0.2955	0.0239	0.0516
327	rs1982441	8	T	G	0.183	0.0316	0.0605
328	rs17405819	8	T	C	0.716	0.0822	0.052
329	rs10971709	9	T	C	0.1839	-0.002	0.0604
330	rs1412235	9	G	C	0.6246	-0.0802	0.0486
331	rs7024334	9	T	G	0.1856	-0.0289	0.06
332	rs10984756	9	C	G	0.91807	-0.0865	0.0843
333	rs7025938	9	G	C	0.3865	0.0148	0.048
334	rs9408882	9	G	A	0.5219	0.0243	0.047
335	rs10867256	9	T	C	0.6178	-0.0572	0.0483
336	rs7869771	9	A	C	0.786	0.0351	0.0575
337	rs4740619	9	C	T	0.5129	0.0635	0.0465
338	rs1928295	9	T	C	0.5026	-0.0237	0.0468
339	rs7871866	9	G	C	0.8047	-0.0539	0.0598

340	rs12098284	10	C	T	0.93296	0.0042	0.0934
341	rs12779328	10	C	T	0.8043	0.184	0.0589
342	rs10795422	10	G	A	0.7142	-0.0059	0.0518
343	rs1624134	10	G	C	0.5325	-0.0898	0.0467
344	rs2163188	10	G	C	0.5277	0.0433	0.0468
345	rs7899106	10	G	A	0.6195	0.0363	0.0483
346	rs7084454	10	G	A	0.6962	-0.0725	0.0507
347	rs12762034	10	T	C	0.8863	-0.0199	0.0735
348	rs1937683	10	T	C	0.6649	0.0628	0.0498
349	rs17113297	10	C	T	0.7658	-0.0087	0.0553
350	rs17636031	10	C	T	0.1522	-0.0486	0.0653
351	rs999889	10	A	G	0.2444	0.0619	0.0546
352	rs10887578	10	C	G	0.4465	0.064	0.047
353	rs577525	10	C	T	0.5032	0.0327	0.0469
354	rs7102454	11	C	T	0.3015	0.0255	0.0508
355	rs7358465	11	C	T	0.2736	0.0098	0.053
356	rs2605603	11	G	A	0.4797	0.1172	0.0467
357	rs10750215	11	T	G	0.3804	0.05	0.048
358	rs2065418	11	T	G	0.691	0.0558	0.0504
359	rs7117238	11	G	A	0.8016	-0.0131	0.0588
360	rs4929923	11	T	C	0.3434	-0.0019	0.049
361	rs4237643	11	G	T	0.691	-0.0569	0.0506
362	rs11030618	11	T	C	0.5462	-0.0003	0.047
363	rs7124681	11	C	A	0.6046	0.0483	0.0501
364	rs1465900	11	C	A	0.2082	-0.0841	0.0573
365	rs1784460	11	T	A	0.7094	-0.0483	0.0516
366	rs2429150	12	C	A	0.46	-0.0521	0.0471
367	rs10878946	12	T	C	0.6462	0.0391	0.0487
368	rs705704	12	A	G	0.3177	0.0579	0.0501
369	rs7488867	12	T	C	0.3144	-0.0462	0.0503
370	rs2306537	12	G	A	0.2629	0.0241	0.0532
371	rs11115176	12	C	T	0.2579	-0.0045	0.0535
372	rs1843328	12	A	C	0.5	-0.0118	0.047
373	rs7318817	13	C	T	0.388	0.0765	0.0482
374	rs1927790	13	T	C	0.4989	-0.0467	0.0466
375	rs6561943	13	C	T	0.7277	-0.0662	0.0526
376	rs9300422	13	G	A	0.7238	-0.0397	0.0522
377	rs7334078	13	C	T	0.2493	-0.0279	0.054
378	rs12429545	13	G	A	0.8615	-0.0891	0.0677
379	rs9538162	13	C	T	0.3862	-0.0188	0.048
380	rs1144387	13	G	C	0.4536	0.0609	0.0472
381	rs1330052	13	G	C	0.4418	0.0218	0.047
382	rs3007105	14	T	C	0.3238	0.062	0.0497

383	rs4981693	14	G	A	0.3329	0.0224	0.0497
384	rs226000	14	T	C	0.7767	0.0537	0.0562
385	rs9989141	14	T	C	0.6269	-0.0149	0.0483
386	rs12888955	14	G	A	0.3709	0.0426	0.0482
387	rs12593036	15	A	G	0.7182	-0.0002	0.052
388	rs11855853	15	T	C	0.2128	-0.0169	0.0574
389	rs7172627	15	G	A	0.4518	0.0031	0.047
390	rs13329567	15	C	T	0.8391	0.1102	0.0634
391	rs12905439	15	G	C	0.345	-0.0233	0.0493
392	rs9806742	15	G	A	0.05813	0.0964	0.0996
393	rs12914489	15	A	G	0.1395	-0.0231	0.0678
394	rs8027205	15	C	G	0.5483	0.0189	0.047
395	rs12448257	16	G	A	0.8427	-0.0978	0.0641
396	rs2361988	16	T	C	0.7916	-0.0052	0.0575
397	rs1896767	16	G	A	0.5051	-0.0238	0.0468
398	rs4786903	16	A	G	0.2419	0.0067	0.0547
399	rs7196720	16	T	C	0.4788	0.063	0.0467
400	rs3814883	16	C	T	0.5961	0.06	0.0475
401	rs12933482	16	A	G	0.93932	-0.072	0.0981
402	rs7206608	16	C	G	0.6596	-0.109	0.0491
403	rs7498665	16	A	G	0.5871	-0.0564	0.0475
404	rs4516268	17	A	C	0.2558	0.045	0.0537
405	rs4968656	17	G	A	0.3068	0.061	0.0506
406	rs12939549	17	G	A	0.3562	-0.0991	0.0489
407	rs2411182	17	G	A	0.246	0.0358	0.0546
408	rs962273	17	T	C	0.2643	-0.1071	0.0528
409	rs1075901	17	T	C	0.4452	-0.0559	0.047
410	rs7222349	17	G	A	0.7028	-0.0305	0.0508
411	rs11656076	17	G	A	0.7391	-0.0617	0.0537
412	rs12602912	17	C	T	0.7633	-0.0931	0.055
413	rs8097672	18	T	A	0.1667	0.0028	0.0629
414	rs1430387	18	C	T	0.5068	-0.0208	0.0467
415	rs4800191	18	C	G	0.6457	0.0047	0.0491
416	rs559231	18	T	G	0.3871	0.0068	0.0482
417	rs663129	18	A	G	0.1821	0.0921	0.0602
418	rs1241986	18	G	A	0.8331	0.0458	0.0625
419	rs7239575	18	C	T	0.4779	-0.0568	0.0468
420	rs1158805	18	C	A	0.5538	0.0252	0.0472
421	rs9951619	18	T	G	0.3485	-0.0066	0.0491
422	rs1982725	19	C	T	0.5357	0.0002	0.0468
423	rs11084553	19	A	G	0.8471	0.0181	0.065
424	rs11672660	19	T	C	0.2259	-0.0385	0.0564
425	rs10408324	19	C	T	0.6944	-0.0135	0.0505

426	rs12981256	19	G	A	0.5713	0.0856	0.0479
427	rs6011457	20	T	A	0.5796	0.0182	0.0473
428	rs1321432	20	C	A	0.7118	-0.0349	0.0514
429	rs2143253	20	G	A	0.8997	-0.037	0.078
430	rs2425840	20	A	C	0.6613	-0.0139	0.0487
431	rs8123881	20	G	A	0.2376	0.114	0.055
432	rs1409818	20	T	C	0.93506	0.0812	0.0952
433	rs17806379	20	C	T	0.8444	0.0568	0.0644
434	rs427943	21	A	C	0.4371	0.0133	0.047
435	rs13047416	21	G	C	0.5021	-0.0698	0.0469
436	rs2836964	21	T	C	0.7666	-0.0278	0.0554
437	rs4818225	21	A	G	0.3814	0.0415	0.0482
438	rs175165	22	T	G	0.5701	0.0357	0.0474
439	rs9615905	22	T	C	0.4357	0.0461	0.0472
440	rs11538	22	A	G	0.7905	0.0244	0.0577
441	rs4820408	22	T	G	0.4015	0.0717	0.0477

Supplementary Table S2. List of genetic instruments for trunk fat mass by each instrumental SNPs.

No.	SNP	chr	EA	OA	EAF	β	SE
1	rs12679106	8	T	G	0.7347	-0.0724	0.0529
2	rs56203712	4	G	A	0.2537	0.062	0.0537
3	rs11782341	8	A	G	0.7777	-0.0755	0.0563
4	rs33503	3	G	A	0.1909	0.0204	0.06
5	rs62190394	2	T	C	0.3149	-0.1063	0.0502
6	rs12619178	2	T	C	0.4063	-0.0615	0.0476
7	rs768023	6	G	A	0.4234	-0.0509	0.0475
8	rs2954021	8	G	A	0.5394	-0.0287	0.0469
9	rs1927626	9	C	T	0.0459	-0.1469	0.1129
10	rs9861443	3	A	C	0.3359	-0.0157	0.0498
11	rs3853252	6	G	A	0.5841	0.0377	0.0477
12	rs16934748	10	C	T	0.1586	-0.0039	0.0636

13	rs3803286	14	A	G	0.3338	0.0603	0.0495
14	rs704061	12	C	T	0.3767	0.0085	0.0482
15	rs1834144	18	C	A	0.5538	0.0252	0.0472
16	rs72976986	19	A	G	0.1589	-0.0097	0.064
17	rs73142879	20	C	T	0.8421	0.0679	0.0641
18	rs2371767	3	C	G	0.2206	-0.0322	0.0562
19	rs7649970	3	C	T	0.8313	-0.0008	0.0624
20	rs6840236	4	T	C	0.5426	-0.0326	0.0468
21	rs7697556	4	C	T	0.508	-0.0557	0.0468
22	rs55672437	4	C	G	0.1733	0.1252	0.0618
23	rs2744965	6	C	T	0.7984	-0.0125	0.0595
24	rs7442885	5	C	G	0.8162	0.0617	0.0601
25	rs13156484	5	A	G	0.5127	0.0338	0.0469
26	rs13209968	6	G	C	0.4912	0.0543	0.0466
27	rs28636067	5	A	G	0.0885	0.1503	0.0819
28	rs12339822	9	A	G	0.4829	-0.0306	0.047
29	rs854917	6	T	C	0.754	0.0157	0.0545
30	rs2482704	9	G	T	0.6242	-0.0209	0.0486
31	rs60898782	6	G	A	0.7182	0.0404	0.0523
32	rs1652376	18	G	T	0.5171	0.0337	0.0468
33	rs33836	19	T	C	0.4654	-0.0113	0.0472
34	rs6029180	20	G	A	0.2807	-0.058	0.0521
35	rs245775	5	G	A	0.6723	0.0464	0.0499
36	rs8087074	18	G	T	0.7539	-0.0157	0.0549
37	rs2287214	12	A	G	0.6216	0.0501	0.0481
38	rs7171864	15	G	A	0.2984	0.1245	0.0512
39	rs11603783	11	C	T	0.1909	0.0781	0.0591
40	rs34560402	11	T	C	0.03518	0.1694	0.1285
41	rs11856579	15	A	G	0.2128	-0.0169	0.0574
42	rs11030119	11	G	A	0.6699	-0.0086	0.0498

43	rs10264581	7	G	A	0.0868	-0.1047	0.0839
44	rs1653892	7	T	C	0.2947	-0.0196	0.0513
45	rs9320823	6	C	T	0.6755	-0.0538	0.0495
46	rs34517439	1	C	A	0.876	-0.1019	0.0714
47	rs6135562	20	C	T	0.06081	-0.0895	0.0979
48	rs7959830	12	G	T	0.6154	0.0333	0.0479
49	rs4477562	13	C	T	0.8615	-0.0891	0.0677
50	rs750090	4	C	T	0.416	0.0094	0.0478
51	rs6717858	2	C	T	0.3747	0.0159	0.0481
52	rs55726687	12	A	G	0.2116	0.0542	0.057
53	rs79535757	12	T	A	0.003611	-0.287	0.3984
54	rs13340461	6	T	C	0.2594	0.0694	0.0532
55	rs314279	6	A	C	0.875	0.0536	0.0704
56	rs58862095	7	C	T	0.4845	-0.0321	0.0468
57	rs11782074	8	T	G	0.36	0.0321	0.0485
58	rs11917068	3	C	T	0.2288	-0.0399	0.0555
59	rs56361700	2	C	T	0.02365	-0.0806	0.1539
60	rs66679256	4	T	C	0.366	0.0747	0.0484
61	rs952227	2	G	A	0.6593	0.0859	0.0494
62	rs252749	5	G	A	0.8162	0.1529	0.0601
63	rs40071	5	C	T	0.2108	0.0165	0.0577
64	rs8178882	8	C	T	0.92468	-0.0718	0.088
65	rs7460093	8	G	A	0.5522	0.0438	0.0472
66	rs2500406	20	A	C	0.3139	0.0051	0.0501
67	rs6598540	15	A	G	0.7501	-0.0189	0.0538
68	rs72718281	4	C	T	0.5927	-0.0255	0.0475
69	rs477895	11	C	T	0.1561	0.0952	0.0642
70	rs17522122	14	G	T	0.5486	0.0014	0.0474
71	rs1491905	14	T	C	0.4127	0.0484	0.0477

72	rs217667	14	C	T	0.8151	-0.0474	0.0604
73	rs11208779	1	C	G	0.5662	-0.0144	0.0475
74	rs9814633	3	G	A	0.6266	-0.0313	0.0483
75	rs6549970	3	T	C	0.5604	0.0073	0.0472
76	rs62106258	2	C	T	0.02104	0.1066	0.1638
77	rs12992672	2	A	G	0.838	-0.0094	0.0635
78	rs543874	1	G	A	0.1763	0.0625	0.0614
79	rs6486057	11	A	T	0.3142	0.0319	0.0505
80	rs4757144	11	A	G	0.506	-0.0233	0.0467
81	rs2979655	8	G	T	0.1323	-0.0178	0.0692
82	rs7305790	12	C	A	0.2473	0.0717	0.0542
83	rs274677	5	T	C	0.3793	-0.0729	0.0482
84	rs62396185	6	G	C	0.5919	0.0541	0.0482
85	rs59738707	5	A	G	0.1793	-0.0495	0.0608
86	rs62407569	6	A	G	0.8166	-0.0182	0.06
87	rs7613261	3	T	A	0.2276	0.065	0.0557
88	rs10863753	1	G	A	0.4328	0.0348	0.0476
89	rs6699744	1	A	T	0.3553	0.0468	0.0491
90	rs12475388	2	G	A	0.5406	-0.0087	0.0467
91	rs1470050	2	T	A	0.3407	0.0147	0.0492
92	rs11023199	11	A	G	0.6124	-0.0145	0.0484
93	rs6973656	7	A	G	0.6393	-0.042	0.0487
94	rs10499014	6	G	C	0.2632	-0.0257	0.0535
95	rs9843653	3	T	C	0.607	0.0386	0.0479
96	rs4673617	2	T	C	0.2598	-0.0134	0.053
97	rs76345589	3	C	G	0.95019	0.0527	0.1083
98	rs241460	1	A	G	0.3806	0.0059	0.048
99	rs2181375	1	G	A	0.596	-0.0052	0.0476
100	rs61813293	1	T	G	0.1488	0.0115	0.0661

101	rs7313748	12	C	T	0.6357	0.0457	0.0488
102	rs4804312	19	T	C	0.4579	-0.0143	0.0473
103	rs9584855	13	T	G	0.7462	0.0142	0.0537
104	rs12578952	12	A	G	0.7083	0.0444	0.0514
105	rs11150745	17	G	A	0.2779	-0.0693	0.0522
106	rs2243928	13	G	C	0.5955	0.0018	0.0477
107	rs62104483	19	A	G	0.3587	-0.0337	0.0491
108	rs11642015	16	C	T	0.5889	-0.0969	0.0475
109	rs16967461	16	T	G	0.06592	-0.0176	0.0937
110	rs1013293	1	G	A	0.6279	0.1048	0.0484
111	rs6696828	1	G	C	0.7989	0.0065	0.0585
112	rs75641275	1	C	A	0.2286	0.0297	0.0554
113	rs11205303	1	C	T	0.3212	-0.0741	0.0509
114	rs846545	1	T	C	0.5087	0.0267	0.0468
115	rs3806251	1	A	T	0.8289	-0.0403	0.0618
116	rs12144626	1	C	T	0.5924	-0.0799	0.0474
117	rs17024393	1	C	T	0.05397	0.1554	0.102
118	rs112646560	1	C	T	0.8105	-0.0002	0.0597
119	rs12724928	1	C	T	0.3538	0.0727	0.0489
120	rs815163	1	C	T	0.4734	-0.066	0.0468
121	rs2678204	1	T	G	0.7036	0.0193	0.051
122	rs2494196	1	C	A	0.6946	0.098	0.051
123	rs4482463	2	A	C	0.9586	-0.0019	0.1178
124	rs4549080	2	C	T	0.686	-0.0553	0.0504
125	rs713586	2	C	T	0.4192	0.013	0.0474
126	rs13416004	2	T	C	0.4385	0.1168	0.047
127	rs72820274	2	A	G	0.4534	-0.0111	0.047
128	rs67536595	2	C	T	0.7878	0.0558	0.057
129	rs10172196	2	A	G	0.3565	0.0008	0.0489
130	rs2216931	2	A	C	0.7373	-0.0168	0.053

131	rs12616638	2	A	G	0.501	-0.0405	0.0468
132	rs6545714	2	A	G	0.6072	-0.0241	0.0476
133	rs1446585	2	A	G	0.7087	-0.0567	0.0519
134	rs72917533	2	C	T	0.1404	0.0719	0.0677
135	rs1454687	3	G	C	0.5909	0.0588	0.0476
136	rs1568489	3	A	G	0.3906	0.0545	0.048
137	rs13067187	3	A	G	0.1821	0.0735	0.0606
138	rs12330631	3	T	C	0.3408	-0.0232	0.0496
139	rs8192675	3	C	T	0.2679	-0.0583	0.0527
140	rs62246314	3	A	G	0.06214	-0.0285	0.098
141	rs56254492	3	T	C	0.2542	-0.0622	0.054
142	rs73213484	4	T	A	0.1265	0.0767	0.0702
143	rs7696324	4	G	A	0.6302	0.0414	0.0488
144	rs6861649	5	C	T	0.6381	-0.0009	0.0486
145	rs11135450	5	G	A	0.5435	-0.0028	0.0472
146	rs2914231	5	C	G	0.1356	-0.0852	0.0683
147	rs4958702	5	T	C	0.3592	0.0211	0.0485
148	rs1503527	5	T	C	0.5751	0.0725	0.0476
149	rs2307111	5	T	C	0.5834	0.0479	0.0476
150	rs2012027	5	T	C	0.7344	-0.1019	0.0532
151	rs12213070	6	A	G	0.386	-0.0742	0.0481
152	rs16880854	6	A	G	0.199	-0.0033	0.0586
153	rs147451284	6	C	G	0.96117	0.059	0.1205
154	rs72995085	6	C	T	0.2538	-0.0272	0.0536
155	rs962554	6	T	C	0.74	0.0596	0.0533
156	rs34045288	6	C	T	0.6676	-0.0821	0.0496

157	rs287834	6	T	C	0.5062	-0.0061	0.0468
158	rs4722398	7	T	C	0.93038	0.1195	0.0911
159	rs1182199	7	C	A	0.6642	-0.0305	0.0495
160	rs10269774	7	A	G	0.3885	0.0671	0.0478
161	rs10237306	7	G	T	0.6093	-0.0324	0.0478
162	rs10100245	8	G	A	0.468	-0.0072	0.0471
163	rs11778934	8	G	C	0.6699	-0.0866	0.0498
164	rs10760724	9	C	T	0.5244	0.0933	0.0467
165	rs1627536	9	T	A	0.4023	0.011	0.0475
166	rs17218879	9	C	G	0.6979	0.0137	0.051
167	rs10756714	9	G	A	0.4762	0.0483	0.0465
168	rs17770336	9	C	T	0.6246	-0.0801	0.0486
169	rs35344761	9	C	A	0.8414	-0.0679	0.0643
170	rs10756798	9	T	C	0.6659	-0.0345	0.0497
171	rs10887578	10	C	G	0.4465	0.064	0.047
172	rs7080838	10	T	C	0.5949	0.0226	0.0475
173	rs2172131	10	C	T	0.49	-0.109	0.0467
174	rs11012732	10	G	A	0.3019	0.0694	0.0508
175	rs2002023	10	C	T	0.5416	-0.0526	0.0469
176	rs10999460	10	C	T	0.7307	-0.0559	0.0524
177	rs1782508	11	G	C	0.6711	-0.0609	0.0498
178	rs7124681	11	C	A	0.6046	0.0483	0.0501
179	rs7933085	11	A	G	0.4981	-0.0316	0.0467
180	rs35060985	11	G	A	0.6918	-0.0584	0.0506
181	rs73041988	11	T	G	0.8629	0.0083	0.0679
182	rs2374947	12	G	A	0.7455	-0.0713	0.0534
183	rs12367809	12	C	T	0.6191	0.0187	0.0483
184	rs59066241	12	G	T	0.1664	-0.0289	0.0628

185	rs882378	12	A	C	0.6283	-0.0442	0.0483
186	rs12877270	13	G	A	0.459	-0.0546	0.0472
187	rs17055384	13	C	T	0.8553	0.0864	0.067
188	rs1336486	13	G	T	0.4233	-0.0577	0.0473
189	rs7987928	13	A	G	0.8029	0.0265	0.0583
190	rs1441264	13	A	G	0.6742	-0.0724	0.0501
191	rs4981693	14	G	A	0.3329	0.0224	0.0497
192	rs2370982	14	C	T	0.763	-0.0465	0.0549
193	rs1427325	14	A	C	0.6632	-0.0256	0.0491
194	rs6575340	14	A	G	0.631	-0.0248	0.0485
195	rs16951304	15	C	T	0.1609	-0.1102	0.0634
196	rs62025831	15	T	C	0.678	0.0952	0.0497
197	rs55637757	16	T	C	0.2158	0.0267	0.0571
198	rs62037365	16	C	G	0.5871	-0.0564	0.0475
199	rs862320	16	C	T	0.5634	-0.022	0.047
200	rs13333747	16	C	T	0.1679	0.0106	0.0636
201	rs3814883	16	C	T	0.5961	0.06	0.0475
202	rs7218014	17	T	C	0.7632	-0.0864	0.055
203	rs35537311	17	T	C	0.388	0.0591	0.0479
204	rs6567160	18	C	T	0.1825	0.09	0.0601
205	rs7238896	18	A	G	0.8327	-0.0046	0.0629
206	rs9964122	18	G	A	0.3715	0.1132	0.0486
207	rs10423928	19	A	T	0.2259	-0.0385	0.0564
208	rs12459368	19	G	A	0.2375	-0.022	0.0551
209	rs7258722	19	A	T	0.4593	0.0431	0.0469
210	rs12974458	19	C	T	0.5713	0.0856	0.0479
211	rs2903738	19	T	A	0.2384	-0.0831	0.0552
212	rs7259070	19	C	T	0.564	0.0048	0.0473
213	rs11538	22	A	G	0.7905	0.0244	0.0577
214	rs4821764	22	G	A	0.4013	0.0115	0.0476

Supplementary Table S3. List of genetic instruments for WC by each instrumental SNPs.

No.	SNP	chr	EA	OA	EAF	β	SE
1	rs2033529	6	G	A	0.3277	0.0822	0.0498
2	rs2293576	11	A	G	0.2705	0.0422	0.0535
3	rs7138803	12	A	G	0.3807	-0.0077	0.0483
4	rs2820292	1	A	C	0.4478	-0.0866	0.0467
5	rs11165623	1	G	A	0.5778	0.0479	0.0473
6	rs6755502	2	C	T	0.838	-0.0092	0.0635
7	rs806794	6	A	G	0.5822	0.0582	0.0481
8	rs943005	6	C	T	0.8008	0.004	0.0586
9	rs2112347	5	T	G	0.5834	0.0479	0.0476
10	rs10938397	4	A	G	0.5255	0.0214	0.0468
11	rs10132280	14	C	A	0.6489	0.0663	0.049
12	rs7239883	18	G	A	0.4304	-0.014	0.047
13	rs929641	2	G	A	0.3801	0.071	0.0481
14	rs16894959	6	C	T	0.2021	0.0049	0.0592
15	rs9400239	6	T	C	0.3696	-0.0406	0.0487
16	rs10767658	11	G	C	0.6655	0.0029	0.0497
17	rs10968576	9	A	G	0.6246	-0.0809	0.0486
18	rs7144011	14	G	T	0.763	-0.0465	0.0549
19	rs7550711	1	C	T	0.94672	-0.134	0.1029
20	rs3127553	1	G	A	0.4272	-0.0017	0.0471
21	rs633715	1	C	T	0.1759	0.0617	0.0615
22	rs6545714	2	A	G	0.6072	-0.0241	0.0476
23	rs3849570	3	A	C	0.4486	0.0172	0.0468
24	rs2325036	3	A	C	0.5649	0.0046	0.0472
25	rs2489623	6	A	C	0.4481	0.0301	0.0469
26	rs6163	10	A	C	0.3769	0.0704	0.0482
27	rs10840100	11	G	A	0.6445	-0.0107	0.0487
28	rs12429545	13	G	A	0.8615	-0.0891	0.0677
29	rs749671	16	A	G	0.3827	0.0564	0.0481
30	rs1558902	16	T	A	0.5965	-0.1008	0.0477
31	rs2531992	16	G	A	0.8201	0.0074	0.0606
32	rs7498665	16	A	G	0.5871	-0.0564	0.0475
33	rs6567160	18	C	T	0.1825	0.09	0.0601
34	rs16996700	20	C	T	0.7705	0.0293	0.0555
