# Supplementary material for: Genetically predicted body fat mass and distribution with diabetic kidney disease: A two-sample Mendelian randomization study
Source: Front Genet. 2022 Sep 29;13:872962. doi: 10.3389/fgene.2022.872962 (PMC9557077; doi:10.3389/fgene.2022.872962)
Supplement: Supplementary file 1 [file DataSheet2.PDF]

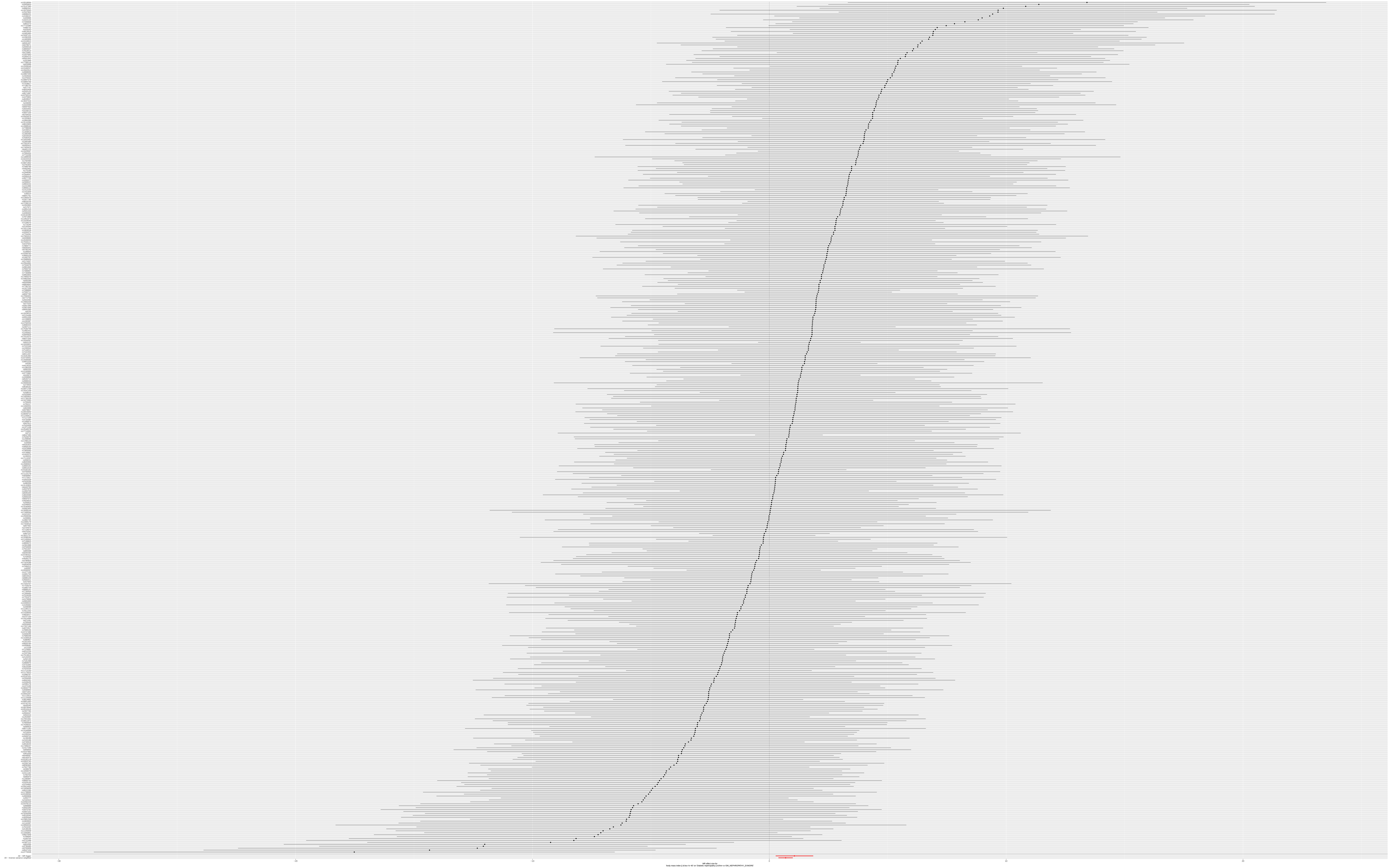

All - MR Egger
All - Inverse variance weighted
-30
-20
-10
0
10
20
MR effect size for
'body mass index || id:ieu-b-40' on 'Diabetic nephropathy || id:finn-a-DM_NEPHROPATHY_EXMORE'

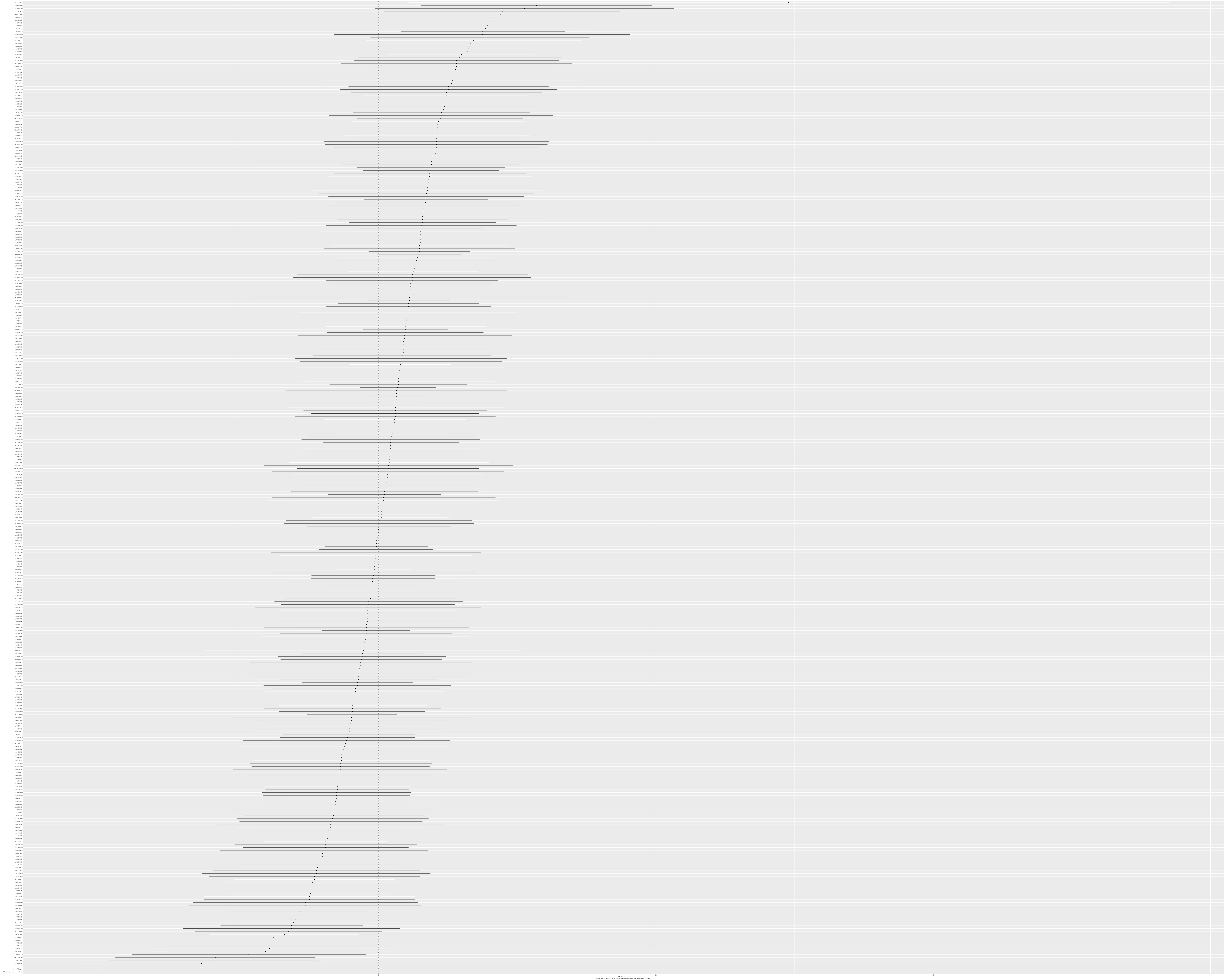

All − MR Egger
All − Inverse variance weighted
−20
0
20
MR effect size for
'Trunk fat mass || id:ukb−b−20044' on 'Diabetic nephropathy || id:finn−a−DM_NEPHROPATHY'

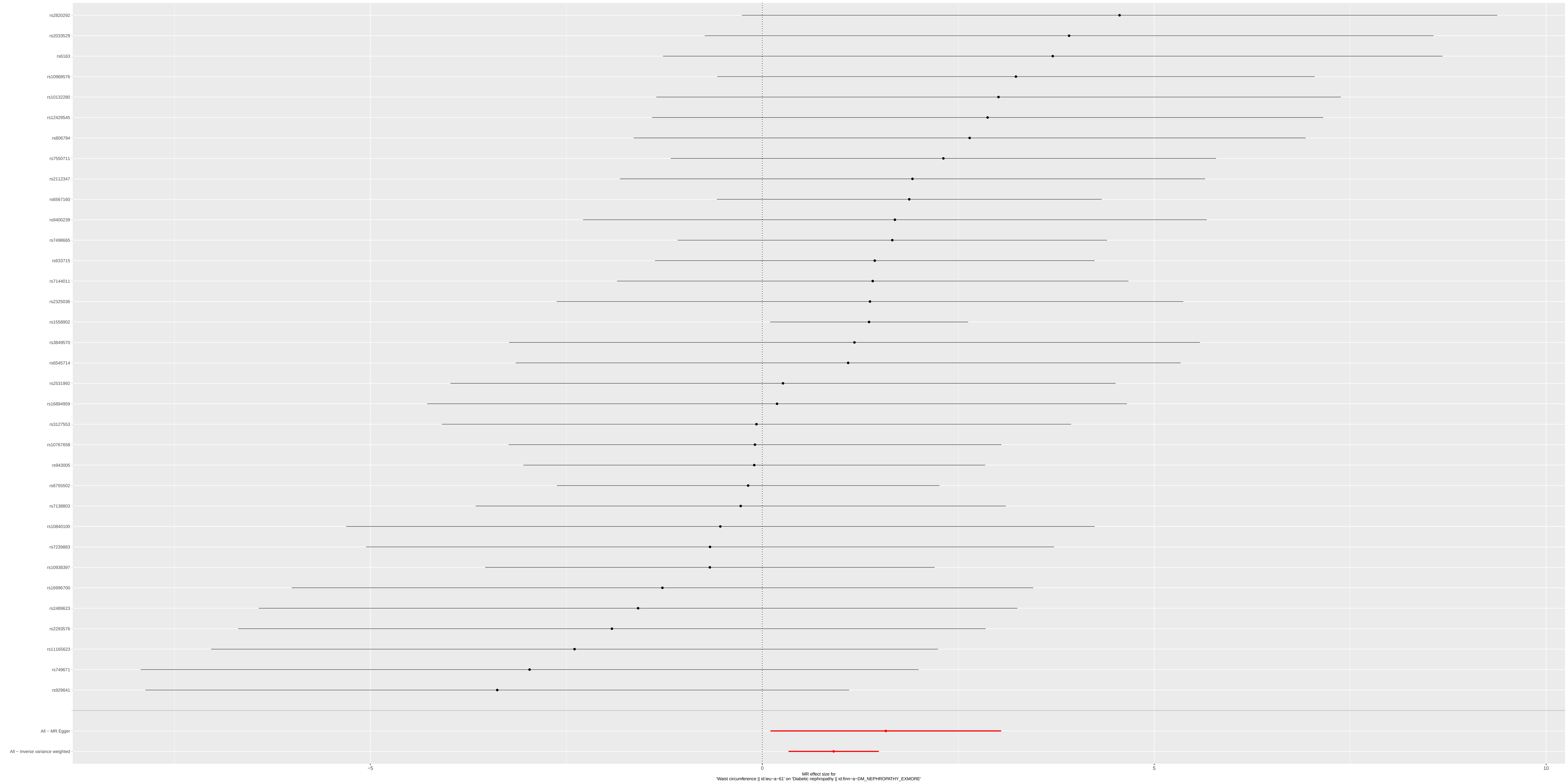
rs2820292
rs2033529
rs6163
rs10968576
rs10132280
rs12429545
rs806794
rs7550711
rs2112347
rs6567160
rs9400239
rs7498665
rs633715
rs7144011
rs2325036
rs1558902
rs3849570
rs6545714
rs2531992
rs16894959
rs3127553
rs10767658
rs943005
rs6755502
rs7138803
rs10840100
rs7239883
rs10938397
rs16996700
rs2489623
rs2293576
rs11165623
rs749671
rs929641
All − MR Egger
All − Inverse variance weighted
−5
0
5
10
MR effect size for
'Waist circumference || id:ieu−a−61' on 'Diabetic nephropathy || id:finn−a−DM_NEPHROPATHY_EXMORE'
